# Plant ecophysiological processes in spectral profiles: perspective from a deciduous broadleaf forest

**DOI:** 10.1007/s10265-021-01302-7

**Published:** 2021-05-10

**Authors:** Hibiki M. Noda, Hiroyuki Muraoka, Kenlo Nishida Nasahara

**Affiliations:** 1 Earth System Division, National Institute for Environmental Studies, 16-2 Onogawa, Tsukuba, Ibaraki 305-8506 Japan; 2 River Basin Research Center, Gifu University, 1-1 Yanagido, Gifu, 501-1193 Japan; 3 Faculty of Life and Environment Sciences, University of Tsukuba, 1-1-1 Tennohdai, Tsukuba, Ibaraki 305-8572 Japan

**Keywords:** Leaf optical properties, Phenology, Radiative transfer model, Satellite remote sensing

## Abstract

The need for progress in satellite remote sensing of terrestrial ecosystems is intensifying under climate change. Further progress in Earth observations of photosynthetic activity and primary production from local to global scales is fundamental to the analysis of the current status and changes in the photosynthetic productivity of terrestrial ecosystems. In this paper, we review plant ecophysiological processes affecting optical properties of the forest canopy which can be measured with optical remote sensing by Earth-observation satellites. Spectral reflectance measured by optical remote sensing is utilized to estimate the temporal and spatial variations in the canopy structure and primary productivity. Optical information reflects the physical characteristics of the targeted vegetation; to use this information efficiently, mechanistic understanding of the basic consequences of plant ecophysiological and optical properties is essential over broad scales, from single leaf to canopy and landscape. In theory, canopy spectral reflectance is regulated by leaf optical properties (reflectance and transmittance spectra) and canopy structure (geometrical distributions of leaf area and angle). In a deciduous broadleaf forest, our measurements and modeling analysis of leaf-level characteristics showed that seasonal changes in chlorophyll content and mesophyll structure of deciduous tree species lead to a seasonal change in leaf optical properties. The canopy reflectance spectrum of the deciduous forest also changes with season. In particular, canopy reflectance in the green region showed a unique pattern in the early growing season: green reflectance increased rapidly after leaf emergence and decreased rapidly after canopy closure. Our model simulation showed that the seasonal change in the leaf optical properties and leaf area index caused this pattern. Based on this understanding we discuss how we can gain ecophysiological information from satellite images at the landscape level. Finally, we discuss the challenges and opportunities of ecophysiological remote sensing by satellites.

## Introduction

In recent decades, climate change has progressed owing to anthropogenic emission of greenhouse gasses, such as CO_2_, and its effect on ecosystems has been apparent from local to global scales (e.g., Gatti et al. 2019; Stöckli and Vidale 2004; Walther et al. 2002). The terrestrial ecosystem is a large carbon sink that absorbs about 30% of anthropogenic CO_2_ via vegetation photosynthesis (Friedlingstein et al. 2019). Photosynthesis is fundamental to all other ecosystem processes and functions, including primary production for the food chain, carbon and energy cycles, and finally climate regulation (Chapin et al. 2011). Photosynthesis and vegetation growth are sensitive to environmental conditions over a broad temporal scale from minutes to seasons and years, and over spatial scales from single-leaf to individual plants and plant communities (Osmond and Chow 1988). The methods to measure photosynthesis and the carbon cycle vary with these scales. Ecological mechanisms of the primary productivity of vegetation and their relationship with meteorological conditions have been studied by biometric surveys (Fang et al. 2014; Gough et al. 2008; Ohtsuka et al. 2007). Micrometeorological measurements of CO_2_ flux from towers allow us to observe the dynamic CO_2_ exchange between the atmosphere and vegetation surfaces (Baldocchi et al. 2001; Owen et al. 2007; Saigusa et al. 2005; Yamamoto et al. 1999). Observations with these methods, however, have been limited to the in situ observation sites which researchers can access physically. The impact of climate change on ecosystems differs across geographic locations, and the relationship between meteorological conditions and ecosystems varies with time. Therefore, we need a method to repeatedly observe structure and functions of ecosystems located in remote places such as mountainous landscapes. Remote sensing by satellite is a powerful tool to observe ecosystems over large spatial and temporal scales. Data obtained by Earth-observation satellites has been widely used to monitor spatial and temporal variations in ecosystem functions and structure (Field et al. 1995; Running et al. 2004; Ustin et al. 2004).

Several ‘vegetation indices’, such as the normalized vegetation index (NDVI) and the enhanced vegetation index (EVI), are used to monitor vegetation (Bannari et al. 1995; Tucker 1979; Wang et al. 2005). The vegetation indices are calculated from remotely sensed spectral reflectance of the vegetation surface which is strongly determined by the biophysical and biochemical characteristics of the vegetation, such as leaf shape and area, leaf pigments and water contents, the amount of non-photosynthetic organs (i.e., branches and stems), and their geometrical distribution. Leaf biochemical components are directly linked to the photosynthetic processes and the canopy geometrical structure determines the light environment within the canopy, and hence these leaf and canopy-level components determine photosynthetic production of the whole canopy (Kitajima et al. 2005). In the growing needs of optical remote sensing to measure the dynamics of canopy structure and functions such as primary productivity in a changing environment, it is essential to mechanistically understand the consequences between the biochemical and structural characteristics and optical properties from single leaf to canopy and landscape scales.

**Fig. 1:**
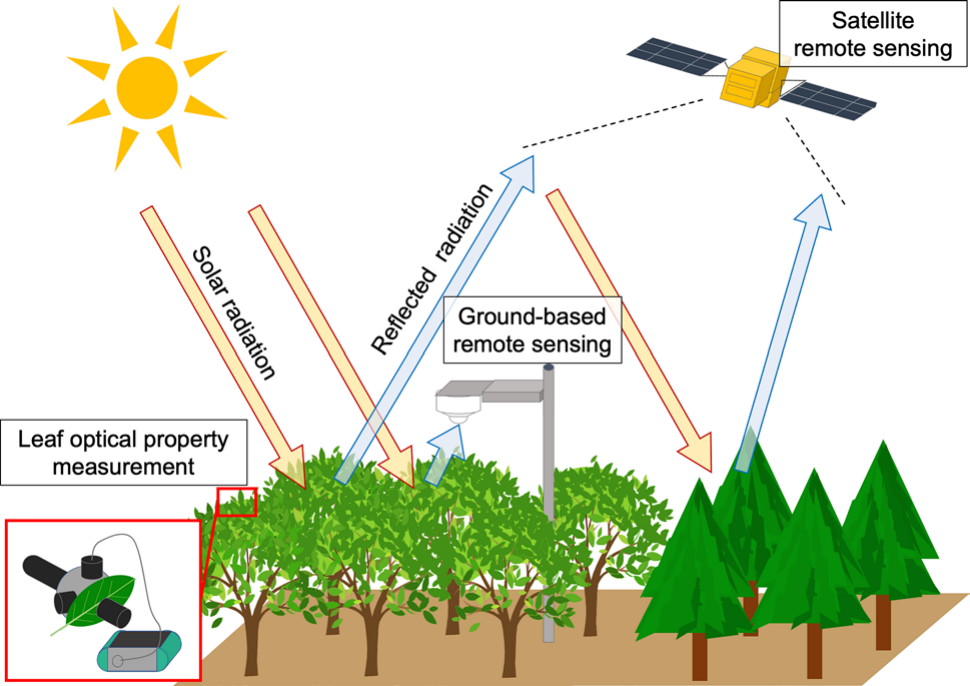
Scheme of multi-scale measurements of optical data at individual-leaf, canopy, and landscape scales

In this paper, we review the relationships between optical and physiological properties across scales from the individual leaf to the landscape. Optical measurements can be conducted at a range of scales: in single leaves using an integrating sphere and a spectrometer, in whole canopies using a tower-mounted spectrometer, and across large landscapes using satellite sensors (Fig. 1). We focused on the seasonality of a deciduous broadleaf forest at Takayama, where long-term and multidisciplinary studies on the carbon cycle have been conducted since 1993 (see Muraoka et al. 2015 for details of the research focus and publications). Takayama is located on a mountainous landscape in a cool-temperate region in central Japan and the forest is dominated by oak and birch. Since this deciduous forest shows a remarkable seasonal change in both single-leaf properties and canopy structure, and hence canopy reflectance spectrum, it is a good example with which to understand the consequences of their changes. On the basis of this understanding, we then show a case of interpreting satellite data from a mountainous landscape where canopy characteristics and environmental conditions are spatially variable.

## Relationships between plant ecophysiological processes and spectral profiles across scales

**Fig. 2:**
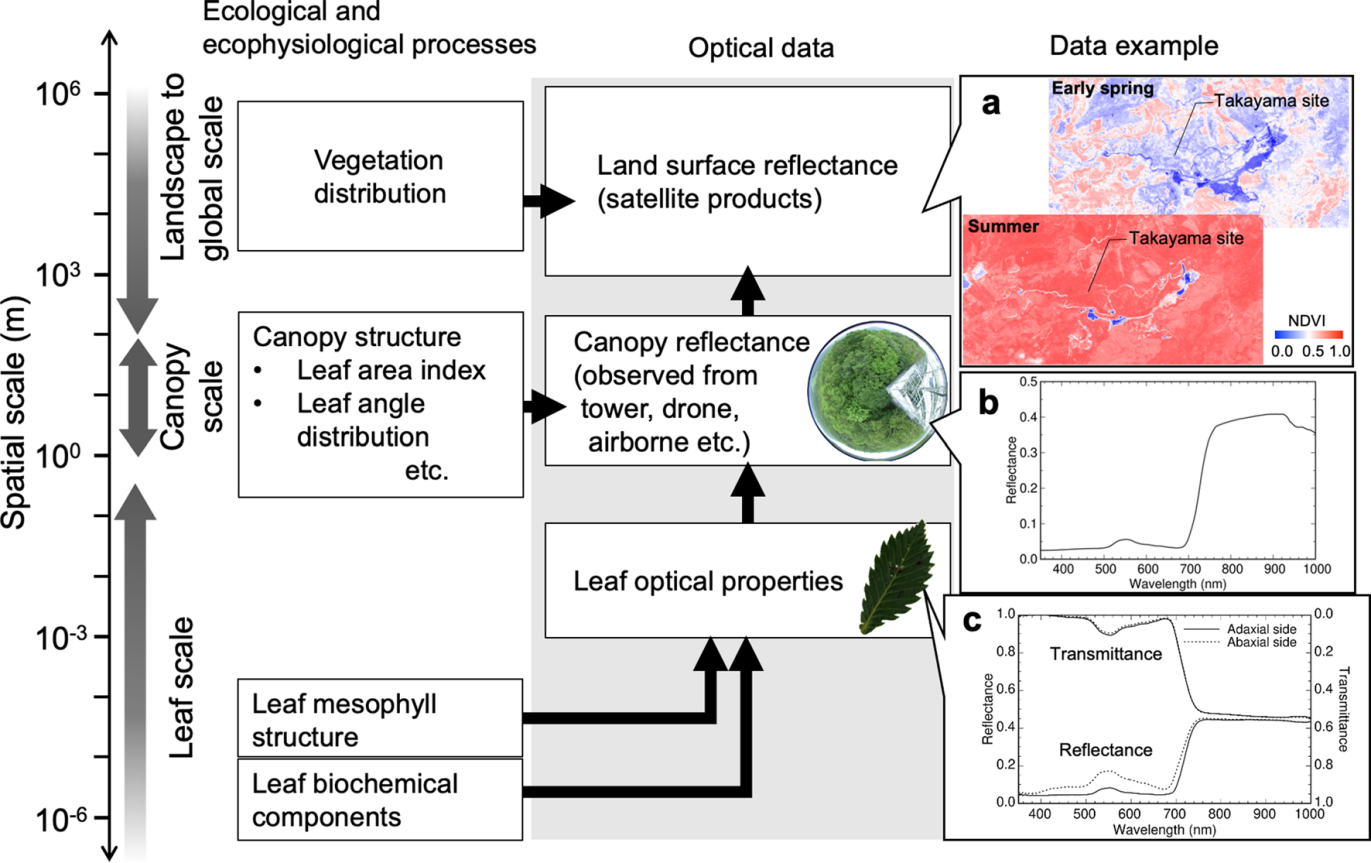
Scaling of ecological and ecophysiological processes and remote sensing studies discussed in this paper. Data examples are an NDVI map around the Takayama site produced by a RapidEye satellite in early spring (15 May 2010; upper panel of **a**) and summer (19 July 2010; lower panel of **a**); canopy reflectance of the Takayama site from a spectroradiometer mounted on a tower on 10 July 2005 (**b**); and the leaf optical properties of *Quercus crispula* at the Takayama site on 10 July 2005 (**c**)

To understand the mechanisms that link optical data—i.e., spectral profiles—to ecophysiological processes in a forest ecosystem, a bottom-up approach from the single-leaf scale would be effective, because smaller-scale phenomena determine the larger scale. Figure 2 shows the scales of ecological and ecophysiological processes, optical data types, and relevant examples.

### Single-leaf scale

The relationship of spectral profiles to physiological properties at the single-leaf scale is fundamental to interpreting remotely sensed vegetation data from the ecophysiological and biophysical perspectives. The leaf optical properties (i.e., reflectance, transmittance, and absorptance spectra) are determined by radiation scattering at the air–water interface and absorption by biochemical components, such as chlorophylls, carotenoids, anthocyanins, lignin, and water, in epidermal and mesophyll cells (Gates et al. 1965; Vogelmann 1993).

**Fig. 3:**
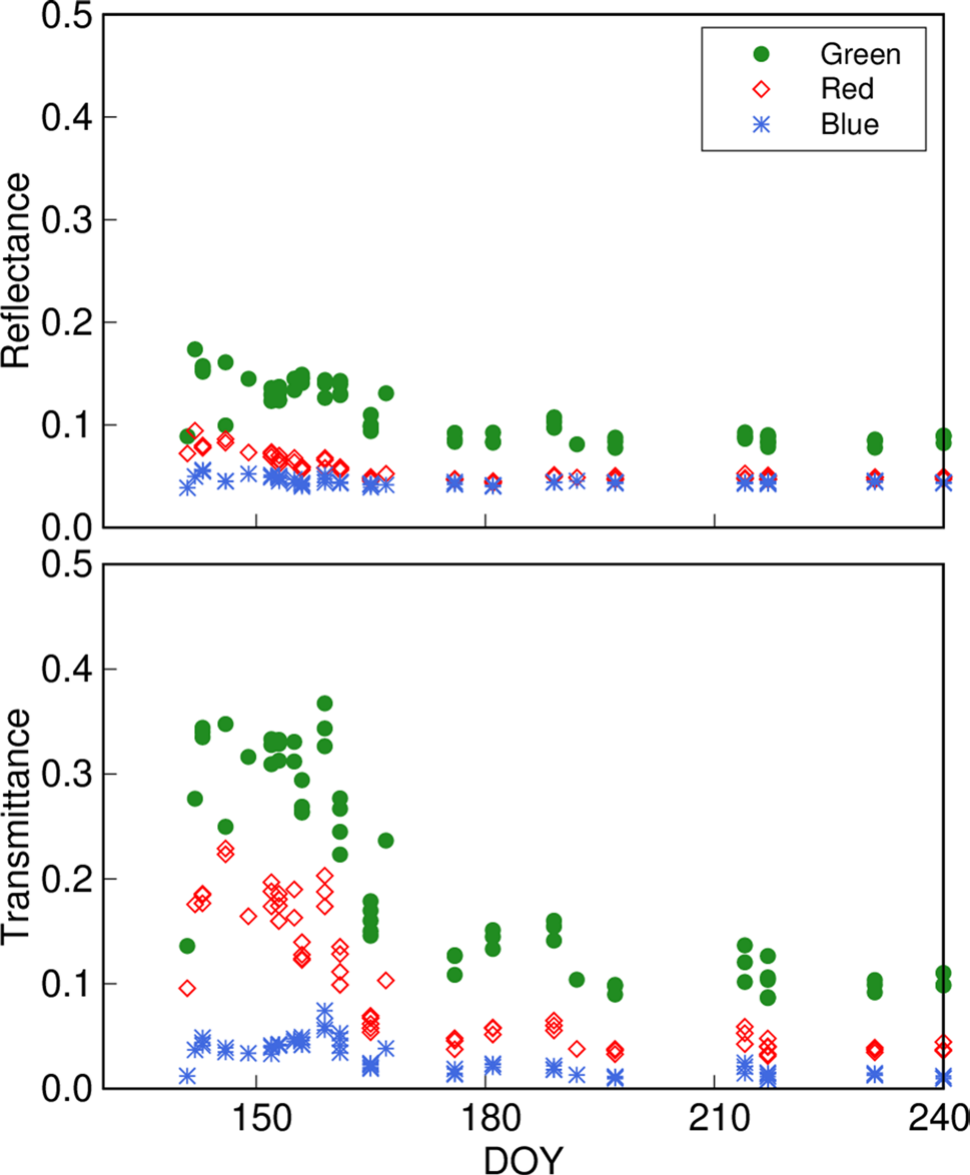
Time series of observed leaf reflectance (**a**) and transmittance (**b**) in green (545–565 nm), red (620–670 nm) and blue regions (459–479 nm) for *Quercus crispula* in Takayama site in 2004–2006 and 2010. Values are data from individual leaf (redrawn from Noda et al. 2021)

**Fig. 4:**
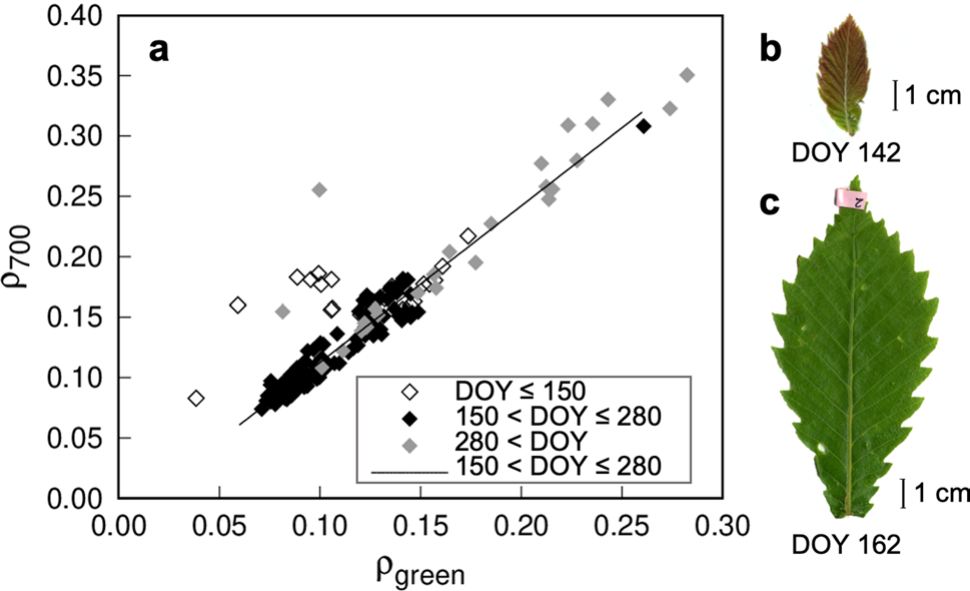
Relationship between leaf reflectance in the green region (545–565 nm; ρ_green_) and at 700 nm (ρ_700_) (**a**), and photos of *Quercus crispula* leaves just after leaf unfolding (**b**) and a few weeks later (c). Data points in the graph are for a single leaf observed in the Takayama site in 2005. The leaves were divided into three groups according to the date (DOY, day of the year): DOY ≤ 150, young leaves; 150 < DOY ≤ 280, mature leaves; 280 < DOY, senescing leaves. Liner regression between ρ_green_ and ρ_700_ for mature leaves is shown

Pigment contents strongly affect the overall spectral patterns in the photosynthetically active radiation (PAR) region (400–700 nm). Chlorophylls have strong absorbance peaks in the red and blue regions of the spectrum (Sims and Gamon 2002; Ustin and Gamon 2010). Carotenoids have a strong absorbance peak in the blue region (400–500 nm), and epidermal flavonoids absorb UV-A (315–400 nm; Burchard et al. 2000). Noda et al. (2021) measured optical properties of leaves at the canopy top (ca. 14 m above the ground) of oak (*Quercus crispula* Blume) and birch (*Betula ermanii* Cham.) in the Takayama site and showed that both reflectance and transmittance in the blue region are always low, even in very young leaves, which have little chlorophyll (Fig. 3). The relatively low chlorophyll content is also sufficient to saturate the absorptance in red region. On the other hand, the reflectance and transmittance in the green region (ca. 550 nm, slightly shorter than the red region) are highly sensitive to chlorophyll content (Gitelson and Merzlyak 1994a, b). In deciduous trees, leaf chlorophyll content increases rapidly during the leaf development period, as shown by Noda et al (2015). Noda et al. (2021) showed that the temporal changes in the transmittance and reflectance are larger in the green region than in the red and blue regions during that period: the reflectance in red and green regions decreased by 39% and 46% and transmittance in those regions decreased by 72% and 80%, respectably, from DOY 143 to 197 in 2004 in *Q. crispula* (Fig. 3). While the green region is largely correlated with the amount of chlorophyll, the spectral pattern in the longer-wavelength region than the red region—the so-called red-edge region (ca. 700 nm)—is commonly used to estimate chlorophyll content (Gitelson and Merzlyak 1998; Gitelson et al. 1996; Sims and Gamon, 2002). Since an anthocyanin-rich leaf has low green reflectance due to an anthocyanin absorption peak in that region, the red-edge region is a more reliable indicator (Merzlyak et al. 2008). Figure 4a shows the relationship between reflectance in the green region (ρ_green_) and that at 700 nm (ρ_700_), the most sensitive wavelength in the red-edge region, in *Q. crispula* from leaf unfolding to leaf fall in 2005 at Takayama (part of dataset published in Noda et al. 2021). We divided these leaf data into three groups according to leaf growth periods; very young (day of year; DOY ≤ 150), mature (150 < DOY ≤ 280), and senescence leaves (DOY > 280). Although ρ_green_ and ρ_700_ of mature leaves showed a linear relationship, those of young leaves tended to be out of the regression line for mature leaves. Since very young *Q. crispula* leaves are often brown because they are rich in anthocyanins (Fig. 4b), their ρ_green_ may be lower than that of leaves with less anthocyanins. Some senescing leaves were also out of the line because of anthocyanin. This is the basic ecophysiological background of several empirical models used for predicting chlorophyll content from leaf reflectance in the red-edge region (Gitelson and Merzlyak 1998; Gitelson et al. 1996; Sims and Gamon, 2002).

The mesophyll structure also affects the leaf optical properties over the entire wavelength range. Especially in the near-infrared (NIR) region (ca. 750–900 nm), where no leaf biochemical component absorbs the radiation, the leaf optical properties are determined mainly by the mesophyll structure. By studying 41 species, Slaton et al. (2001) found that reflectance in the NIR (ρ_NIR_) is positively correlated with the ratio of the surface area of the mesophyll cells exposed to intercellular air space per unit leaf surface area. The phenological changes in ρ_NIR_ and transmittance in NIR (τ_NIR_) of deciduous broadleaf trees also help us to understand the consequences of those with mesophyll structure: young leaves just after unfolding have low ρ_NIR_ and high τ_NIR_, and then ρ_NIR_ increases and τ_NIR_ decreases rapidly during leaf development (Demarez et al. 1999; Noda et al. 2021). In general, the initial stage of leaf development is characterized by packed and small mesophyll cells. After leaf unfolding, the mesophyll cell volume and intercellular air space expand rapidly (Miyazawa and Terashima 2001; Niinemets et al. 2012; Sims and Pearcy 1992; Tichá 1985). With such developmental changes of mesophyll structure, ρ_NIR_ and τ_NIR_ also change. The optical properties in the PAR region are also affected by the mesophyll structure but their phenological patterns are not simple because of the effect of chlorophyll (Noda et al. 2021). While development of mesophyll structure increase reflectance in the spectral region of PAR (ρ_PAR_) and decrease transmittance in the region (τ_PAR_), increase of leaf chlorophyll decrease both ρ_PAR_ and τ_PAR_. Thus, the effects of these two factors on ρ_PAR_ cancel each other out, and hence, ρ_PAR_ decreased little during the leaf development period. On the other hand, development in mesophyll structure and leaf chlorophyll content both led to decreases in τ_PAR_, and hence, τ_PAR_ decreased rapidly.

These relationships can be quantitatively examined by mathematical analysis incorporating a radiative transfer theory. PROSPECT (Jaquemoud and Baret 1990; Jacquemoud et al. 2009) is the most popular radiative transfer model for broadleaf species. This model is based on the ‘plate model’ proposed by Allen et al. (1969), and the mesophyll tissue is assumed to be a pile of compact opaque layers. The model specifies the average number of air/cell wall interfaces within the mesophyll and simulates radiative transfer within the leaf. There are several versions of PROSPECT models with different algorithms. PROSPECT-5 considers the effect of carotenoids to achieve high accuracy of modeling for young leaves (Feret et al. 2008); PROSPECT-D adds the effect of anthocyanins for old leaves (Féret et al. 2017). PROSPECT models do not consider leaf dorsiventrality, whereas some other models do (Stuckens et al. 2009; Ustin et al. 2001; Yamada and Fujimura 1991). A model for conifer needles, LIBERTY (Leaf Incorporating Biochemistry Exhibiting Reflectance and Transmittance Yields), has been developed by Dawson et al. (1998).

### Canopy scale

Incoming radiation is absorbed, transmitted or reflected by leaves and branches in the canopy and by the ground surface. The optical properties of leaves and branches and their geometrical structure, including leaf-area and leaf-angle distributions, strongly determine the light conditions within the canopy which determine the total amount of radiation absorbed by leaves and used for photosynthetic production (Kitajima et al. 2005; Monsi and Saeki 1953; republished in 2005; Reich 2012). The fate of scattered radiation led by transmission and reflection is observed by optical sensors such as a spectroradiometer mounted on an observation tower or Earth observation satellites. Canopy reflectance has been used to estimate canopy structure and photosynthetic production (e.g., Muraoka et al. 2013; Wang et al. 2005). In a deciduous forest, phenological phenomena are most apparently represented by the seasonal changes in leaf area index (LAI) due to leaf emergence, growth, and fall (Muraoka and Koizumi 2005; Mussche et al. 2001; Nagai et al. 2017; Nasahara et al. 2008). Leaf optical properties also show seasonal changes with leaf age, as mentioned above. A combination of these components determines the spectral profile of radiation reflected by vegetation canopies.

**Fig. 5:**
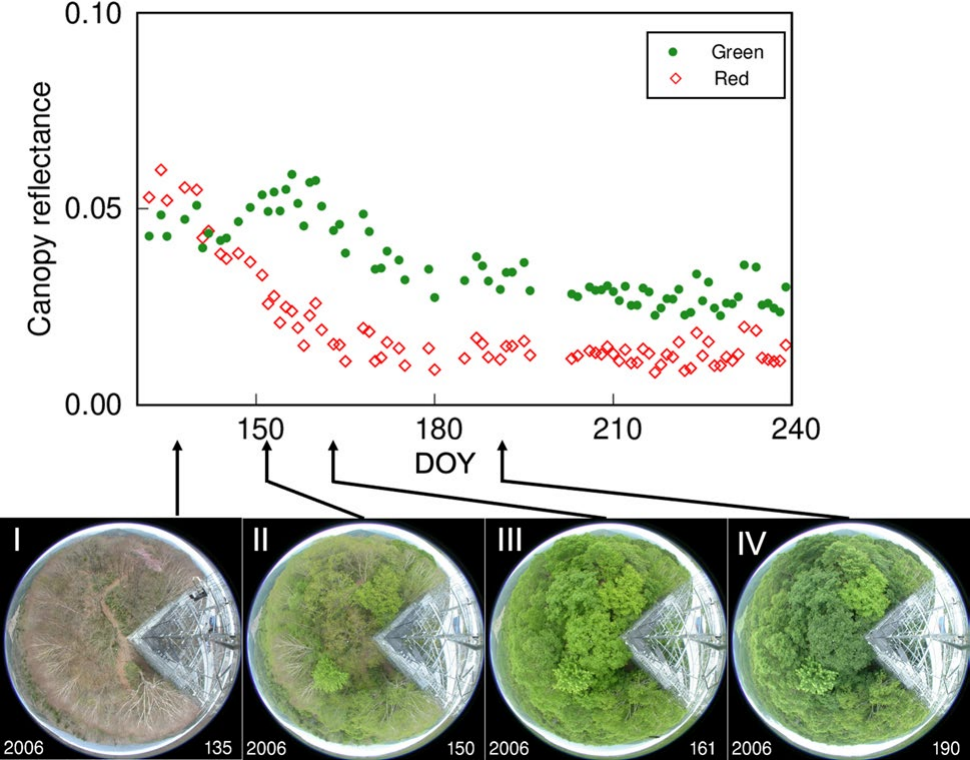
Time series of observed canopy reflectance in the green and red regions and fisheye photographs of the canopy (I–IV) in a deciduous broadleaf forest at the Takayama site in 2006. The numbers in the bottom right corner of each photo are DOY. Green (545–565 nm) and red (620–670 nm) reflectance values were calculated as the ratios of radiation reflected by the canopy to incoming radiation

Canopy reflectance can be observed automatically by a spectroradiometer mounted on a fixed location, such as an observation tower used to monitor CO_2_ flux, at various sites (Gamon et al. 2006; Nasahara and Nagai 2015). Several studies have reported remarkable seasonal changes in canopy reflectance and vegetation indices and their possible consequences with canopy phenology (e.g., Motohka et al. 2010; Muraoka et al. 2013; Nagai et al. 2016; Nakaji et al. 2008). Motohka et al. (2010) observed seasonal patterns in canopy reflectance from four vegetation types—two deciduous forests, a paddy field, and a grassland—in Japan. They showed that the patterns of green and red reflectance of the deciduous forests are unique during the early growing season: green reflectance increased after leaf emergence and decreased after canopy closure, while red reflectance continued to decrease after leaf emergence. Figure 5 shows the seasonal patterns in canopy reflectance in the green and red regions at the Takayama site in 2006. With increasing leaf coverage in the early growing season, red reflectance decreased rapidly and then remained low, but green reflectance increased sharply, peaked at around DOY 160, and then decreased. We were able to identify a bright green color of the forest canopy at the time of the peak of green reflectance (see canopy photographs in Fig. 5). Motohka et al. (2010) also showed that green and red reflectance in the grassland and paddy field were almost constant through the seasons and finally, the authors indicated that the green–red vegetation index (GRVI) would be a good indicator to monitor canopy phenology in deciduous forests, particularly the timing of leaf emergence and fall. How then can we understand the seasonal phenomena in the deciduous forest from the leaf-level optical properties and canopy structure?

**Fig. 6:**
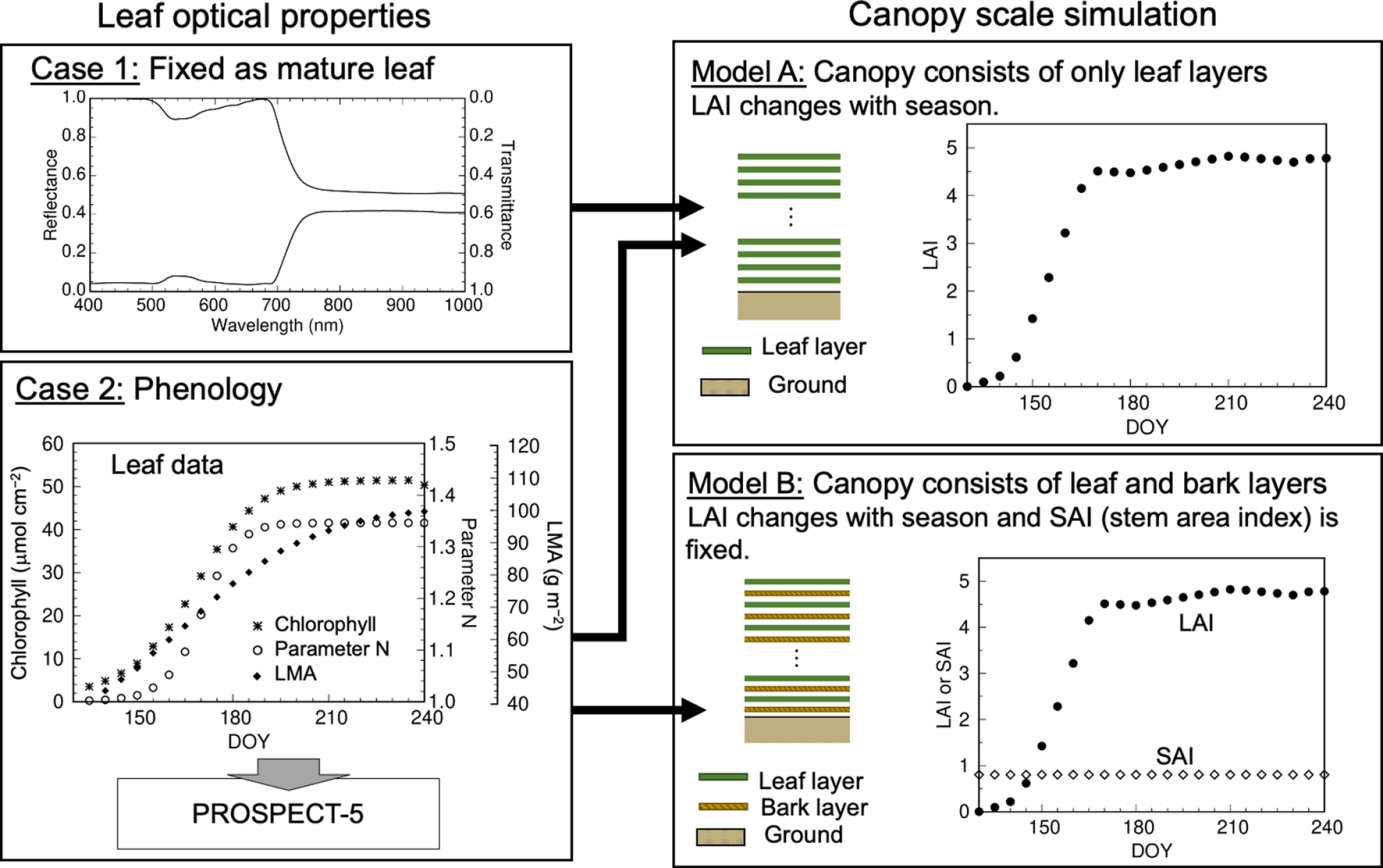
Description of the canopy model used to simulate the phenological patterns in canopy reflectance. “Parameter *N*” in Case 2 is a parameter representative of mesophyll structure for PROSPECT models

**Fig. 7:**
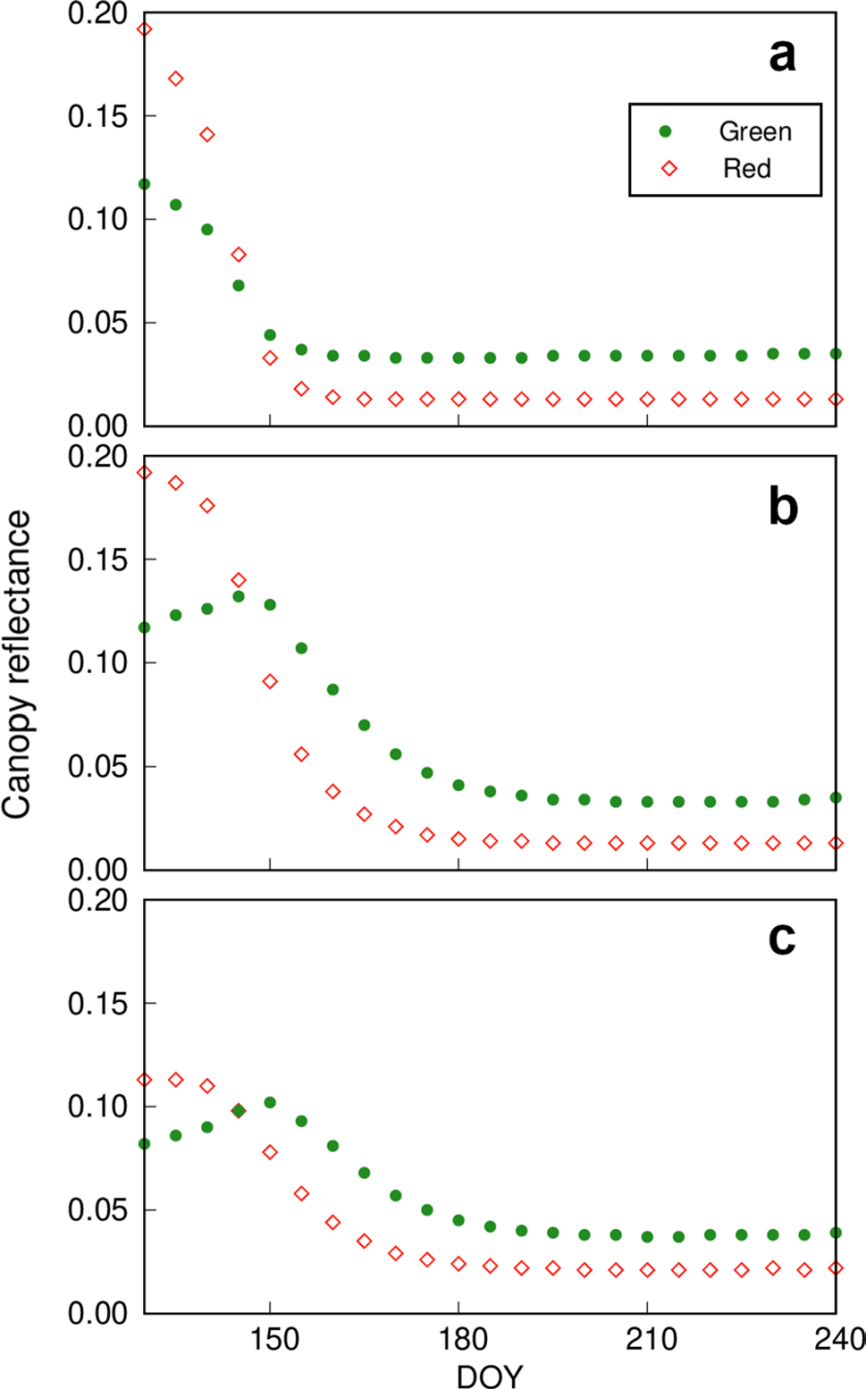
Time series of canopy reflectance in the green and red regions simulated with the ordinal SAIL model (the canopy consists only of leaves; Model A in Fig. 6) (**a**, **b**) and the modified SAIL model (the canopy consists of leaves and branches (Model B in Fig. 6) (**c**). To run the models, the leaf optical properties were fixed as for mature leaves (Case 1 in Fig. 6) (**a**) or changed with the phenological stage of the leaves (Case 2 in Fig. 6) (**b**, **c**)

To examine the consequences of leaf-level optical properties and canopy structure, we combined the data for single leaves and canopy leaf distribution (presented as LAI) over the seasons by using the mathematical radiative transfer model SAIL (Scattering by Arbitrary Inclined Leaves; Verhoef 1984). SAIL is an extension of a 1-D canopy bidirectional model by Suits (1972) and includes a leaf angle distribution to output a more realistic pattern of canopy bidirectional reflectance (Badhwar et al. 1985). Figure 6 shows a schematic diagram of our analysis with SAIL. Figure 7 shows the results of simulation of canopy reflectance in the green and red regions in 2006, the same year as in Fig. 5.

*Model A* This model assumes that the canopy consists of only leaves (no branches or stems), as in the original SAIL model, and considers two cases: (1) leaf optical properties are constant throughout the seasons, and (2) leaf optical properties have seasonal changes (Fig. 6). In both cases, LAI changes seasonally according to long-term field observation data at the Takayama site (Nagai et al. 2017). In this model, since the field observations of LAI were periodical with irregular time intervals, the seasonal data were gap-filled with 5-day steps, assuming linear changes during the leaf development phase and mature phase (Fig. 6). When we calculated the canopy reflectance with the SAIL model assuming constant leaf optical properties (Case 1), as in the middle of the growing season (DOY 200), and seasonal LAI, green and red reflectance was very high at the beginning of the season (DOY 130, timing of snow melt) and dropped sharply after leaf emergence with no peak in green reflectance (Fig. 7a). Then we considered seasonal changes in leaf optical properties (Case 2), which we estimated with PROSPECT-5 by using the phenological patterns in chlorophyll and carotenoid contents, leaf mass per area, and parameter *N* (a parameter representative of mesophyll structure for PROSPECT models) of *Q. crispula* (Noda et al. 2021). In the case of both LAI and leaf optical properties changing with season, both green and red reflectance at the beginning of growing season were high, as in Case 1; then, both dropped sharply again, with a small peak of green reflectance on DOY 145 (Fig. 7b). These results indicate that the canopy reflectance of a deciduous broadleaf forest is determined by a combination of seasonal changes (phenology) of both canopy LAI and leaf optical properties, which are influenced by leaf biochemical components and mesophyll structure. We would also like to highlight a phenomenon in the early growing season (before leaf emergence, DOY < 150). Since the canopy in the original SAIL model consists of leaves only, the effects of tree branches and trunks on radiation scattering and absorption are not considered. However, in the early spring after the snow melt, the branches are not covered by leaves, and the reflectance from those canopy components should be important.

*Model B* To consider the effects of tree branches and trunks (in other words, the “bark” of the canopy), we modified SAIL by incorporating “bark layers” to be inserted between leaf layers (Fig. 6). For the bark layers, the reflectance spectrum of the *Q. crispula* trunk (Noda et al. 2014) was used and the stem area index was fixed as 0.8 according to Nasahara et al. (2008). When seasonal changes in LAI and leaf optical properties were simulated with the modified SAIL model, the patterns of green and red reflectance were more realistic (Fig. 7c). At the time of leaf emergence, red and green reflectance values were lower and the overall patterns were close to the observed ones, with a peak of green reflectance (compare Figs. 5 and 7c). This result suggests that branches and trunks are important components to determine radiation transfer in the canopy and final reflectance in remote sensing.

These findings support our hypothesis that leaf-level optical properties, canopy structure, and their phenology matter in canopy spectral reflectance. However, we also recognize that the estimated green reflectance peaked on DOY 150, which is earlier than the observed timing. The estimated peak coincided with the LAI reaching 1.0, which theoretically means that the ground is entirely covered by one layer of leaves. However, in a real forest, the ground is not completely covered by leaves even if the LAI has reached 1.0 at DOY 150 (see photograph II in Fig. 5). The peak in the real forest occurred around DOY 160, when the ground was covered completely by leaves (see photograph III in Fig. 5). This discrepancy is caused by the heterogeneous distribution of leaves in the canopy (‘leaf clumping’). This is a good example showing that we need to consider all such ecophysiological and ecological backgrounds to properly understand and estimate what the spectral data indicate for canopy ecological processes (see also Muraoka and Koizumi 2009; Muraoka et al. 2010). Focusing on the 3-D architecture of a tree crown, which has species-specific characteristics due to leaf shape and branching pattern, is a key to address the heterogeneity and diversity of canopy structure and function by remote sensing (e.g., Zellweger et al. 2019). Leuzinger and Körner (2007) measured the canopy surface temperature in a temperate forest in Switzerland, and showed that the difference between canopy leaf and air temperatures vary among species and that it was caused by the species-specific combination of canopy architecture and leaf traits (e.g., leaf shape, stomatal conductance). To appraise such 3-D structure of canopies, active remote sensing technique, i.e., laser scanning (also known as light detection and ranging, LiDAR) has progressed in recent decades. Airborne laser scanning maps 3-D structure of the canopy from above and so-called Terrestrial Laser Scanning, TLS, from below the canopy provides extremely detailed information including understory vegetation (García et al. 2015; Hosoi and Omasa 2007; Omasa et al. 2007; Zhu et al. 2018).

### Landscape to global scale

**Fig. 8:**
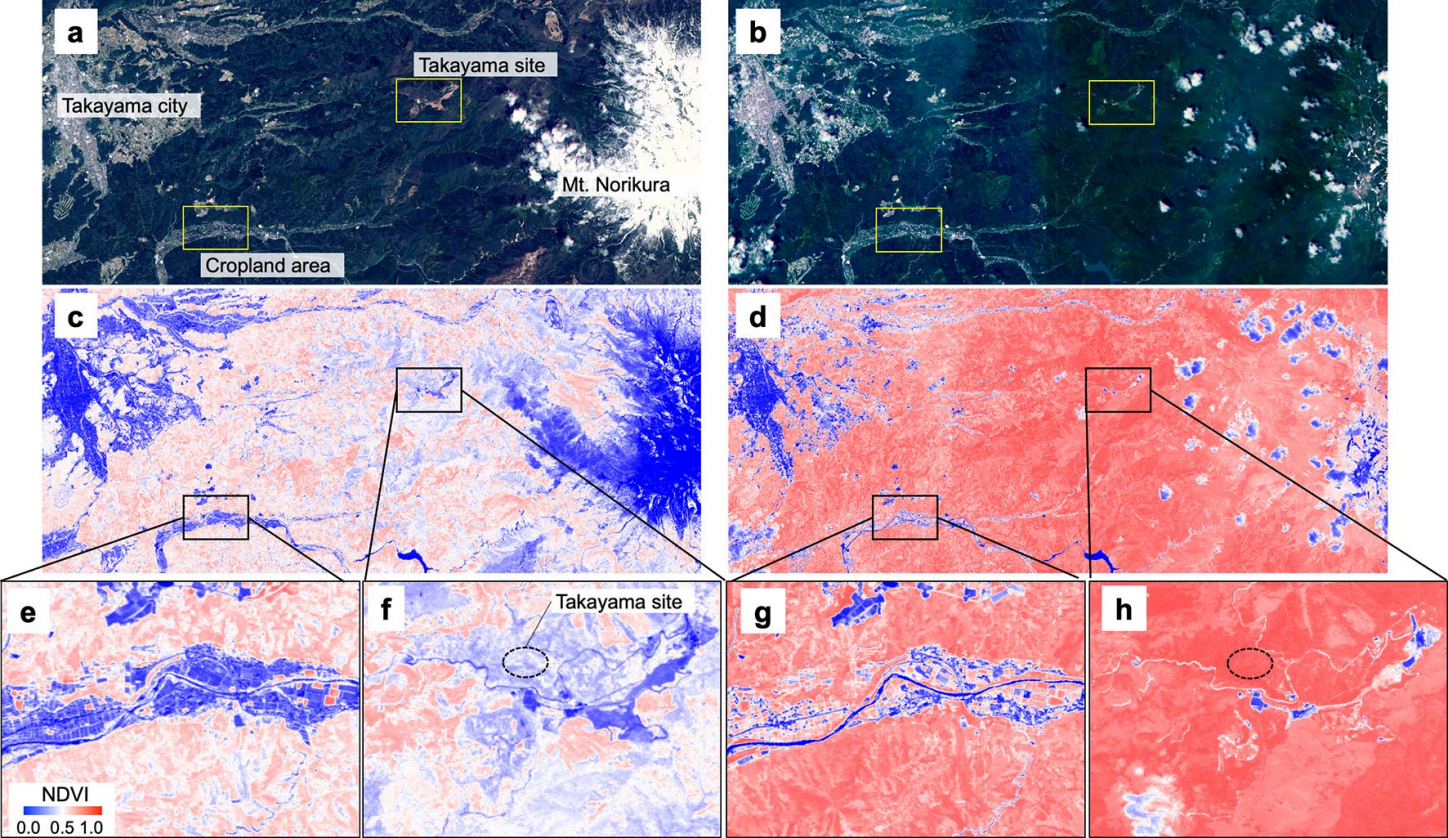
Satellite images (RapidEye) of the Takayama region in true colors (**a**, **b**) and NDVI images (**c**–**h**) taken on 15 May (**a**, **c**, **e**, **f**) or 19 July 2010 (**b**, **d**, **g**, **h**). Magnified NDVI images are shown for a cropland area (**e**, **g**) and around the Takayama site (**f**, **h**)

Advancement of Earth observation satellites enables us to observe spatial scales from ecosystem to landscape, regional, and global scales; the strength of these satellites is their capability of repeated observations of the same locations on Earth (Cavender-Bares et al. 2020; Reed et al. 2003). The satellite data enables us to extend the knowledge from in situ observation data at a research-plot level to landscape to global scales. Cross-scale links help us to understand what the spectral information in the satellite imagery indicates for a given observed area (e.g., landscape). One of the challenges to scale-up the canopy-level in situ remote sensing to landscape-level satellite remote sensing observation for diverse forest ecosystems on a mountainous landscape is the consistency between optical information from these different platforms. In the Takayama site we have shown that spectral reflectance information is useful to scale-up the plot-level canopy ecological characteristics to landscape level, but caution is needed for atmospheric and topographic corrections in addition to ecological understanding in the spectral information as discussed above (Melnikova et al. 2018; Nagai et al. 2010). These careful validations would allow us to gain spatial information of ecosystem structure from satellite imagery. Figure 8 shows true-color images and an NDVI map of the mountainous landscape in which our Takayama site is located, observed by a RapidEye satellite in early spring (15 May 2010) and summer (19 July 2010). The distribution of NDVI values was heterogeneous, particularly in spring (Fig. 8c, e, f). If we know that this landscape consists of different types of vegetation—i.e., deciduous forests, evergreen forests, and croplands (rice paddy fields)—we could interpret the data as follows. The locations with low NDVI in spring (blue in Fig. 8c, e, f) and high NDVI in summer (red in Fig. 8d, g, h) are dominated by deciduous forests, reflecting their remarkable change in leaf area along the phenological phases. On the other hand, the locations with relatively high NDVI in both spring (light red areas in Fig. 8c, e, f) and summer are dominated by evergreen cedar plantations. Additional satellite data in between these two seasons would allow us to observe how the phenology of forests along altitudinal or latitudinal gradients changes (Nagai et al. 2016). Archived satellite data collected over many years enable us to analyze long-term changes in the vegetation response to climate change. For example, by analyzing the Advanced Very High Resolution Radiometer (AVHRR) NDVI data set, Stöckli and Vidale (2004) showed that the phenological trend of vegetation has shifted to earlier (− 0.54 days per year) and prolonged (0.96 days per year) growing periods in the past 20 years in Europe. On the basis of the NDVI data of AVHRR and MODerate resolution Imaging Spectroradiometer (MODIS), Wang et al. (2017) revealed that the weakening of summer monsoon circulation in the past three decades has affected the greening pattern in South Asia.

As mentioned above, there is a growing demand to monitor and detect the effects of the on-going climate change on plant growth, vegetation dynamics, and ecosystem functions such as the carbon cycle in terms of CO_2_ flux, primary production, and carbon sequestration at daily to yearly scales (Cias et al. 2014; Muraoka and Koizumi 2009). One challenge is to precisely measure the photosynthetic ‘activity’ in a plant physiological sense at ecosystem to landscape scales with special attention to the impacts of extreme climatic events (drought, heat stress, and unexpected frost) related to climate change (Reichstein et al. 2013). Another challenge is to precisely observe the temporal (phenological) change of the ecosystem functions, including carbon and water cycles, as they are fundamental to ecosystem services (Piao et al. 2019; Richardson et al. 2013; Tang et al. 2016). In general, two approaches can be used to apply ground-based observations to satellite data. One is to find a correlation between ecosystem phenomena, for example canopy phenology (leaf emergence, maturation and leaf fall) or CO_2_ flux (gross and net primary production), and spectral information such as vegetation indices measured by satellites (e.g., Nagai et al. 2010; Xiao et al. 2004). The other approach is first to examine relationships between the ecosystem phenomena and in situ spectral data obtained at ground observation sites in detail, and then to validate the satellite-derived spectral data with the in situ spectral data for spatial up-scaling. Muraoka et al. (2013) found dynamic relationships between five different kinds of vegetation indices (e.g., NDVI, EVI, chlorophyll index) measured on the tower and daily maximum canopy photosynthetic rate (GPP_max_) throughout seasons at the Takayama site. They then applied the in situ EVI–GPP_max_ relationship to EVI by Terra/MODIS to estimate the spatial and seasonal patterns of GPP in central Japan. But we also recognize that we still have challenges in remote sensing techniques to observe the dynamic ecosystem physiological functions in detail such as ordinary plant ecophysiological studies in a changing environment.

## Recent challenges of remote sensing observations for spatial and temporal dynamics of ecosystems

As discussed above we need to consider several issues in order to scale-up ground-based ecological and physiological knowledge to the broader scales by satellite remote sensing. In this section we discuss the challenges by focusing on spectral features and time resolution of sensors, and then on the retrieval of biochemical information from the spectral data. Challenges related to the spatial scales of biodiversity and ecosystem observations are well discussed in other recent reviews (e.g., Anderson 2018; Muraoka et al. 2012; Pettorelli et al. 2018; Vihervaara et al. 2017).

### Spectral features of sensors for detection of physiological processes

As ecosystem information relies on the spectra that can be measured by sensors (Gamon et al. 2019), spectral resolution is crucial for precise observation of ecophysiological characteristics such as photosynthetic capacity and activity. The satellite sensors widely used for vegetation remote sensing, such as Terra and Aqua MODIS and NOAA AVHRR, measure radiation in several broad wavelength bands. The broad-band vegetation indices—e.g., NDVI and EVI—have been widely used in satellite remote sensing of the geographical distribution of terrestrial vegetation, LAI, and primary productivity (e.g., Wang et al. 2005). These vegetation indices indicate green biomass, which can be converted to ‘photosynthetic capacity’ which is close to the GPP_max_ as demonstrated by Muraoka et al. (2013) as mentioned above.

In addition to these traditional global observations, there is a growing demand and possibility to measure the physiological responses of ecosystems to environment change by satellite remote sensing (Rogers et al 2017; Ustin et al. 2004). The development of satellite vegetation indices for monitoring photosynthetic activity would enable us to observe the photosynthetic responses to climate change and extreme events at landscape to regional scales. To achieve this through the advancement of remote sensing techniques or data analysis algorithms, it would be essential to first find appropriate spectral characteristics for monitoring photosynthesis.

In general, ground observations use continuous spectra with very high resolution (e.g., Asner and Martin 2009; Meroni et al. 2004), but very few hyperspectral satellite sensors are available because of difficulties in system design, data processing, and radiometric calibration (Qi et al. 2012). The difference in the spectra between in situ spectroradiometers and satellite sensors makes it challenging to directly extend ground-based findings to satellite data. For example, application of information obtained from leaf-level chlorophyll fluorescence measurements on the status of photosystem II electron transport activity or of heat dissipation via the xanthophyll cycle (Baker 2008) should be key for transferring knowledge of plant physiology to broad-scale measurements by satellite remote sensing. The Photochemical Reflectance Index (PRI; calculated from the reflectance at 531 and 570 nm) is well known as a sensitive optical index to detect changes in xanthophyll pigments in live leaves, and it can be used to characterize the diurnal xanthophyll cycle response (Gamon et al. 1992; 1997). Hikosaka and Noda (2019) have experimentally shown the feasibility of assessing the quantum yield of photochemistry and photosynthetic rate from the PRI and chlorophyll fluorescence at the individual-leaf scale. To monitor the physiological status and phenology of ecosystems, MODIS is expected to be the most suitable sensor because it has a very high temporal resolution (daily observations). However, because the original PRI bands are not available from MODIS, band 11 (526–536 nm) and band 12 (546–556 nm; Rahman et al. 2004) or band 1 (620–670 nm; Garbulsky et al. 2013; Goerner et al. 2011) have been used instead. While the satellite remote sensing scientists expect to use this “MODIS PRI” to detect environmental stress on ecosystem-scale photosynthesis, Gamon et al. (2016) pointed out that “MODIS PRI” differs spectrally and functionally from the original PRI and is an indicator of the chlorophyll/carotenoid ratio.

Recent advancement of satellite sensors with high spectral resolution has made it possible to perform global measurements of solar-induced chlorophyll fluorescence (SIF). The satellite remote sensing community expects SIF to indicate photosynthetic activity (Porcar-Castell et al. 2014), which should be influenced by solar radiation, temperature, and water availability in the same way as single-leaf photosynthesis. Although chlorophyll fluoresces are very weak under natural conditions, SIF can be detected passively in narrow dark lines of the solar and atmospheric spectrum in which irradiance is strongly reduced (the so-called Fraunhofer lines; Carter et al. 1990, 1996; Plascyk 1975). To obtain accurate SIF values, it is necessary to use a high-spectral-resolution sensor, which can measure Fraunhofer lines.

Ground-based SIF measurements have been well characterized, and it has been shown that SIF is a good indicator of light use efficiency or photosynthetic production (Meroni et al. 2009). To extend this approach to the monitoring of continental vegetation and to map photosynthetic activity in large areas, experimental satellite missions for SIF observation have been proposed several times since the 1990s (Moya et al. 2004; Rascher et al. 2008). The first SIF observation at a global scale by a satellite was achieved by using spectral data from thermal and near-infrared sensor for carbon observation—Fourier transform spectrometer (TANSO-FTS) of Greenhouse gases Observing SATellite (GOSAT), launched in 2009 (Frankenberg et al. 2011; Joiner et al. 2011). The main mission of GOSAT is to measure atmospheric greenhouse gasses (CO_2_ and CH_4_), while band 1 of TANSO-FTS covers the overlapping wavelengths of the solar Fraunhofer lines and chlorophyll fluorescence with high spectral resolution and is thus suitable for SIF retrieval. Lee et al. (2013) have demonstrated that GOSAT SIF measurements over tropical forests show clear water stress signals at midday that are not well represented in traditional vegetation indices such as NDVI or EVI. Other satellite sensors for atmospheric monitoring are available to retrieve SIF, such as MetOp GOME-2 (Global Ozone Monitoring Experiment-2; Joiner et al. 2013), OCO-2 (Orbiting Carbon Observatory-2; Sun et al. 2017), and Sentinel 5-P TROPOMI (TROPOspheric Monitoring Instrument; Köehler et al. 2018). TANSO-FTS2 of GOSAT-2, the successor to GOSAT, is also available to retrieve SIF, and GOSAT-2 SIF has been already released as an official product. FLEX (FLuorescence EXplorer), a satellite aimed mainly at SIF observation, will be launched in 2024 by the European Space Agency. These currently available satellites and future sensors promise to advance the satellite remote sensing of ecosystem physiology such as photosynthesis at landscape, regional, and global scales.

### Time resolution for leaf and canopy phenology

Time-series analysis of satellite data has been used to characterize vegetation dynamics such as succession (Hall et al. 1991), land use change (Hansen et al. 2013), response to environmental stress (AghaKouchak et al. 2015; Reichstein et al. 2007; Saigusa et al. 2010), and phenology (Cleland et al. 2007; Piao et al. 2019; Stöckli and Vidale 2004; Tang et al. 2016). However, in general, the temporal resolution of satellite data is coarse relative to the temporal scale of phenological events or short-term vegetation responses to changing environment. For example, detection of year-to-year changes in phenology caused by global warming would need at least a few days’ interval considering the rapid growth in the early growing season (Zhang et al. 2009). A moderate-spatial-resolution polar-orbiting satellite sensor, like Terra MODIS, observes the same location once a day (at around 10:30 local time in Japan), but the daily satellite data are generally composited into a cloud-free image, which results in a coarse temporal resolution data with mixed information for one to two weeks (Stöckli and Vidale 2004; Zhang et al. 2009).

However, since such satellite sensors provide well calibrated data with large coverage, it is very convenient for vegetation observation. To use the data for phenology monitoring effectively, combined satellite and ground-observation data has been analyzed (e.g., Nagai et al. 2014). In some cases, to detect the phenological events from MODIS data, a time series of vegetation indices is applied to a simple sigmoid function and the dates of the events are estimated mathematically (Ahl et al. 2006; Zhang et al. 2003, 2004).

Several researchers have tried to use a multi-channel imager onboard a geostationary satellite for vegetation remote sensing (Fensholt et al. 2006; Miura et al. 2019). Miura et al. (2019) successfully used the Himawari-8 data to detect phenological patterns of forest ecosystems in Japan and validated the results with in situ phenological observations from automated digital cameras provided by the Phenological Eyes Network. Since a geostationary satellite maintains the same position relative to Earth’s surface, its sensor provides data for the same area with short time intervals, such as 15 min for Himawari-8. Of course, the cloud cover problem remains, but such satellite sensors would help us to observe the temporal changes of ecosystem structure and functions in short intervals. The Geostationary Carbon Cycle Observatory (GeoCarb), a satellite scheduled for launch in 2024, plans to observe SIF from a geostationary orbit (Moore et al. 2018), and will enable monitoring of photosynthetic activity at high temporal frequency.

### Retrieval of ecophysiological characteristics from remotely sensed data

Retrieval of leaf biochemical components (e.g., chlorophyll, nitrogen and water content), LMA and canopy structure parameters (e.g., LAI) from remotely sensed data will enable the ecosystem and Earth system sciences to investigate the diversity of ecosystem functions along climatic gradients and environmental changes from landscape to global scales (Ito et al. 2015; Rogers et al. 2017). Inversion of physical model and empirical approach are available to estimate the biochemical and structural parameters of vegetation canopy.

Inversion of a physical model, i.e., the radiative transfer model, is thought to be robust for estimating the leaf and canopy parameters because it only deals with physical processes which directly connect plant geometrical features and spectral dynamics. PROSAIL (Baret et al. 1992), the coupling of SAIL and PROSPECT models, is one of the most widely used models (Berger et al. 2018; Jacquemoud et al. 2009). Although PROSAIL is a simple 1-D model, it demonstrates reasonable results. Bacour et al. (2002) compared simulated canopy reflectance by PROSAIL and three more complicated models with observed data by POLarization and Directionality of the Earth's Reflectances (POLDER), satellite sensor and showed that PROSAIL agrees with other models well in terms of the simulated reflectance and parameter effects. PROSAIL inversion is not only applied to airborne data (e.g., Jay et al. 2017), but also satellite data, including broad-band sensors such as MODIS (e.g., Zhang et al. 2005) and Landsat (e.g., Bayat et al. 2018). However, such physical models cannot estimate parameters which are not considered in the algorithm, such as leaf lignin and nitrogen.

Empirical approach, which employs empirical regression equations, has been also used to estimate leaf biochemical components (e.g., nitrogen and lignin) from observed spectrum (e.g., Peterson et al. 1988; Wessman et al. 1988; Yin 1992). For canopy reflectance data obtained by a sensor with high wavelength resolution, partial least squares regression (PLSR) is suggested to be useful for estimating leaf properties (Asner and Martin 2009). In PLSR, the full reflectance spectrum is collapsed into a smaller set of independent variables, or factors, with the measured canopy nitrogen used directly during the spectral decomposition process. While PLSR has a potential to provide detailed leaf parameters, this method can only be applied for high spectral resolution data obtained by hyperspectral sensors on a tower, airborne platform (Asner et al. 2009; 2011) or Hyperion instrument of NASA’s Earth Observing-1 (EO-1) satellite (Martine et al. 2008; Ollinger and Smith 2005).

## Future perspectives

In this paper, we review how leaf-level optical properties are tightly coupled to leaf biochemical and anatomical structures, and how the canopy-scale spectral reflectance is driven by single-leaf optical properties and the geometrical structure of leaves and stems. If we could convert spectral data into plant physiological and ecological data such as photosynthesis and its phenology, satellite remote sensing could be further used for ecological research over broad spatial scales in changing environments. Although several problems exist in scaling up ecological and ecophysiological findings at single-leaf and canopy scales to the landscape scale, at which satellite observation is advantageous, we should be able to overcome these problems by accumulating experimental knowledge along biochemical, biophysical, and biogeographical theories as discussed above. The advancement of new satellite sensors which considers critical spectral bands for plant ecophysiology also helps us to apply knowledge of plant ecophysiology to fully use satellite data for ecosystem and biodiversity research. To further develop the satellite remote sensing of vegetation structure and functions, it is necessary to keep up to date with information on both ecophysiology provided by plant scientists and on satellite missions provided by space institutions.
